# Determinants of stunting and severe stunting among under-fives in Tanzania: evidence from the 2010 cross-sectional household survey

**DOI:** 10.1186/s12887-015-0482-9

**Published:** 2015-10-21

**Authors:** Lulu Chirande, Deborah Charwe, Hadijah Mbwana, Rose Victor, Sabas Kimboka, Abukari Ibrahim Issaka, Surinder K. Baines, Michael J. Dibley, Kingsley Emwinyore Agho

**Affiliations:** Muhimbili University of Health and Allied Sciences, Dar es Salaam, Tanzania; Tanzania Food and Nutrition Centre, Dar es Salaam, Tanzania; Sokoine University of Agriculture, Morogoro, Tanzania; School of Health Sciences, University of Newcastle, New South Wales, Australia; Sydney School of Public Health, University of Sydney, New South Wales, Australia; School of Science and Health, Western Sydney University, Building 24.2.40, Campbelltown Campus, Locked Bag 1797, Penrith, NSW 2571 Australia

**Keywords:** Stunting, Under-fives, Deaths, Undernutrition, Tanzania

## Abstract

**Background:**

Stunting is one of the main public health problems in Tanzania. It is caused mainly by malnutrition among children aged less than 5 years. Identifying the determinants of stunting and severe stunting among such children would help public health planners to reshape and redesign new interventions to reduce this health hazard. This study aimed to identify factors associated with stunting and severe stunting among children aged less than five years in Tanzania.

**Methods:**

The sample is made up of 7324 children aged 0-59 months, from the Tanzania Demographic and Health Surveys 2010. Analysis in this study was restricted to children who lived with the respondent (women aged 15-49 years). Stunting and severe stunting were examined against a set of individual-, household- and community-level factors using simple and multiple logistic regression analyses.

**Results:**

The prevalence of stunting and severe stunting were 35.5 % [95 % Confidence interval (CI): 33.3-37.7] and 14.4 % (95 % CI: 12.9-16.1) for children aged 0-23 months and 41.6 % (95 % CI: 39.8-43.3) and 16.1 % (95 % CI: 14.8-17.5) for children aged 0-59 months, respectively. Multivariable analyses showed that the most consistent significant risk factors for stunted and severely-stunted children aged 0-23 and 0-59 months were: mothers with no schooling, male children, babies perceived to be of small or average size at birth by their mothers and unsafe sources of drinking water [adjusted odds ratio (AOR) for stunted children aged 0-23 months = 1.37; 95 % CI: (1.07, 1.75)]; [AOR for severely stunted children aged 0-23 months = 1.50; 95 % CI: (1.05, 2.14)], [AOR for stunted children aged 0-59 months = 1.42; 95 % CI: (1.13, 1.79)] and [AOR for severely stunted children aged 0-59 months = 1.26; 95 % CI: (1.09, 1.46)].

**Conclusions:**

Community-based interventions are needed to reduce the occurrence of stunting and severe stunting in Tanzania. These interventions should target mothers with low levels of education, male children, small- or average-size babies and households with unsafe drinking water.

## Background

Stunting arises as a result of chronic restriction of a child’s potential growth brought about by the cumulative effects of inadequate food intake and poor health conditions that result from endemic poverty [1]. This restricted growth is an important cause of morbidity and mortality in infants and children [2, 3]. Poor socioeconomic conditions and an increased risk of frequent and early exposure to adverse conditions, such as illness or inappropriate feeding practices may give rise to high levels of stunting. A decline in the national stunting rate is usually an indication of improvements in the overall socioeconomic conditions of a country [4]. The global variation of the prevalence of stunting is considerable, ranging from 5 to 65 % among the less-developed countries [5]. In developing countries, the prevalence of stunting starts to rise at about three months of age and then slows at around two years of age [5].

According to Black et al. [3], more than one-third of child deaths and more than 10 % of the total global disease burden are attributed to maternal and child undernutrition, which may result in stunting among others. The global burden of stunting is enormous, with approximately 195 million occurring in the developing world [5]. Many developing countries report far higher rates of stunting prevalence than any other illnesses due to child undernutrition, making it an important public health issue.

Among the different regions of Africa, the decline in stunting has been found to be greatest in the northern and middle parts. However, the prevalence has hardly changed in the other (eastern, western, and southern) sub-regions of the continent [6]. It is estimated that there are presently 171 million stunted preschool children worldwide, of which approximately 98 % reside in developing countries and about 35 % in Africa. Due to expanding population, the number of stunted pre-school children in Africa as a whole increased from 51 million in 2000 to 60 million in 2010, and if present trends do not change, these numbers are reported to further increase to 64 million in 2020 [6].

According to the 2010 Tanzania Demographic and Health Survey (TDHS), 42 % of Tanzanian children aged less than five years are stunted [7] and places Tanzania among the 10 worst-affected countries in the world. In spite of a reduction from 48 % (1996) to 42 % (2010), the prevalence of child stunting in Tanzania in 2010 was still ‘unacceptably high', by World Health Organization (WHO) standards and greater efforts are thus required to decrease the prevalence of stunting among Tanzanian children.

Factors that may indirectly influence stunting levels among children in developing countries include socio-economic status such as mother’s education and occupation, household income and health expenditure [8–10]. In addition, factors such as micronutrient deficiencies, inadequate protein intake and infections may directly cause stunting [11, 12]. There have been several studies on risk factors for stunting from different countries. For instance, a study on the magnitude and determinants of stunting in children aged 5 years or younger in food surplus region of Ethiopia found males, children aged less than 7 months and children who contracted diarrhoea to be significantly more likely to be stunted [13].

Another study on the determinants of linear growth and predictors of severe stunting during infancy in rural Malawi found the risk factors for severe stunting to be: preterm birth (<37 gestational weeks), maternal short stature (<160 cm), maternal failure to gain >200 g/week during pregnancy, home delivery and paternal illiteracy [14]These studies, however, have been limited in scope as they were not population-based. In Tanzania, there have been few recent studies on factors associated with stunting among children. These studies, however, have been limited in scope. For instance, a recent cross-sectional study [15] conducted in Tanzania revealed that low birth weight and low BMI of mothers were the strong predictors of stunting among children. This study covered only one district – the Kilosa district. Another recent Tanzanian cross-sectional study [16] used multivariate logistic regression model to show that maternal education and child’s age were independent predictors of stunting. This study also covered just one district – the *Same* district of the Kilimanjaro region of Tanzania. Thus, there has not been any recent population-based study that has investigated risk factors of stunting in Tanzania. This study therefore aimed to identify and discuss factors associated with stunting and severe stunting among children aged 5 years or younger, using the latest TDHS dataset. Results of this study would contribute to the extant literature and enable policy makers to institute interventions to minimise the burden of stunting and severe stunting in Tanzanian children.

### Ethics

This study was based on an analysis of existing public domain survey datasets that are freely available online with all identifier information removed. The survey was approved by the Ethics Committee of the ICF Macro at Calverton in the USA and by the Ethics Committee in Tanzania. Written consent was obtained from all respondents and all information was collected confidentially.

## Methods

### Data sources

The data examined were from the 2010 Tanzania Demographic and Health Survey (TDHS 2010). The survey involved completed interviews of 10,139 ever-married women aged 15–49 years and utilised three questionnaires: a household, women’s and men’s questionnaire. The survey collected anthropometric data for all sampled children in Tanzania; including those who were not biological off-springs of the women interviewed in the survey. Each trained interviewer carried a scale and measuring board. The scales were lightweight, bathroom-type with a digital screen. Recumbent heights were measured for children aged less than 24 months whilst the standing heights of older children were measured. The present analysis was restricted to the children aged 0–59 months, living with the respondent and alive. The total weighted sample size was 7324, and the survey yielded a response rate of 96.4 %.

To determine their risk factors, the outcome variables (stunting and severe stunting), were examined against a set of individual-, household- and community-level factors. Individual-level factors included variables from attributes of the parents, infant and mother-infant dyad. Household wealth index and source of drinking water constituted the household-level factors while community-level factors were type of residence (urban or rural) and geographical zones.

Household wealth index was calculated as a score of household assets such as ownership of means of transport, ownership of durable goods and household facilities, which was weighted using the principal components analysis method [17]. This index was divided into five categories (quintiles), and each household was assigned to one of these categories. In the TDHS datasets, household wealth index variable was categorized into five quintiles (poorest, poorer, middle, richer and richest).

### Statistical analyses

To determine the level of stunting and severe stunting in children aged 0-23 months and 0-59 months, the dependent variable was expressed as a dichotomous, that is, category 0 (not stunted (>-2SD) or not severely stunted (>-3SD) and category 1 (stunted (>-2SD) or severely stunted (>-3SD).

Analyses were performed using Stata version 12.1 (StataCorp, College Station, TX, USA). ‘Svy’ commands were used to allow for adjustments for the cluster sampling design, sampling weights and the calculation of standard errors. The Taylor series linearization method was used in the surveys to estimate confidence intervals (CIs) around prevalence estimates. The chi-squared test was used to test the significance of associations. Multiple logistic regression was used to adjust for the complex sampling design and weights. Univariate binary logistic regression analysis was performed to examine the association between stunted and severely stunted children aged 0-23 months and overall stunted children aged 0-59 months.

In the multivariable analysis models, a manual procedure of stepwise backward elimination process was used to identify factors that were significantly associated with the study outcomes using 5 % significance level. In order to avoid or minimise any statistical error in our analysis, we repeated the manual procedure of stepwise backward elimination process by using a different approach. This involved three steps: (1) only potential risk factors with *P*-value < 0.20 were entered in the backward elimination process, (2) the backward elimination was tested by including all variables (all potential risk factors); and, (3) Any collinearity was tested and reported in the final model. The odds ratios with 95 % CIs were calculated in order to assess the adjusted risk of independent variables, and those with *P* < 0.05 were retained in the final model.

## Results

### Characteristics of the sample

Of the total sample of 7234 children aged 0-59 months, the majority lived in rural areas (80.3 %). Approximately 84 % of the interviewed mothers were employed in the past 12 months, and 6.2 % had secondary education or higher. Of the total births, 49.7 % took place at a health facility. Only a small proportion of deliveries (4.3 %) took place by caesarean section. Male (49.8 %) and female (50.2 %) children were nearly equally represented in the sample. About 99 % of mothers had made at least one antenatal clinic visit during pregnancy, and 45.2 % of the mothers were aged 25–34 years. About 12 % of children were exclusively breastfed and 47.8 % of children were breastfed in addition to being given supplements. According to the mothers’ perception, 70.6 % of children were of average size, 7.9 % were of small or very small size and 29.5 % were of large size at birth. Nearly 42 % of mothers could not read a sentence. About 21 % of children lived in the Western geographical zone and 20.3 %, 13.9 % and 2.7 % of children lived in the Lake, Southern Highlands and Zanzibar regions respectively (Table 1).

**Table 1:** Characteristics of parents and children aged 0–59 months in Tanzania 2010 (*n* = 7324)

Characteristic	n	%
*Individual level factors*		
*Parental factor*		
Maternal working status		
Non-working	984	13.4
Working (past 12 months)	6340	86.6
Maternal education		
No education	1887	25.8
Primary	4982	68.0
Secondary and above	456	6.2
Partner's occupation		
Non agriculture	2168	29.6
Agriculture	4759	65.0
Not working	398	5.4
Partner's education (*n* = 6932)		
No education	1266	18.3
Primary	5090	73.4
Secondary and above	576	8.3
Mother's age		
15–24 years	2188	29.9
25–34 years	3310	45.2
35-49 years	1826	24.9
Mother's age at birth		
< 19 years	1091	14.9
20–29 years	3805	52.0
30–39 years	2141	29.2
40 and above	288	3.9
Marital status		
Currently married	6260	85.5
Formerly married (div/sep/widow)	701	9.6
Never married	363	5.0
Birth order		
First-born	1439	19.6
2nd -4th	3546	48.4
5 or more	2339	31.9
Preceding birth interval		
No previous birth	1439	19.7
< 24 months	895	12.2
> 24 months	4979	68.1
Place of delivery		
Home	3684	50.3
Health facility	3640	49.7
Mode of delivery (*n* = 7301)		
Non-caesarean	6987	95.7
Caesarean	314	4.3
Type of delivery assistance (*n* = 7193)		
Health professional	3567	49.6
Traditional birth attendant	976	13.6
Relatives and other untrained personnel	2388	33.2
No one	262	3.6
Antenatal clinic visits (*n* = 5134)		
None	98	1.9
1–3.	2839	55.3
4+	2198	42.8
Timing of postnatal check-up (*n* = 7235)		
No check-ups (including missing)	5536	76.5
0–2 days	814	11.2
3–6 days	327	4.5
7 + days	559	7.7
Maternal BMI (*n* = 7240)		
<= 18.5 (kg/m2)	668	9.2
> 18.5 (kg/m2)	6572	90.8
Child breastfeeding (BF) status		
Exclusive BF	840	11.5
BF + water	177	2.4
BF + supplements^a^	3499	47.8
No BF	2809	38.4
Mother is literate (*n* = 7257)		
No	3024	41.7
Yes	4233	58.3
Mother read newspaper (*n* = 7317)		
No	6418	87.7
Yes	900	12.3
Mother listened to the radio (*n* = 7322)		
No	3537	48.3
Yes	3785	51.7
Mother watched TV		
No	6342	86.6
Yes	982	13.4
*Child level factors*		
Sex of baby		
Male	3647	49.8
Female	3678	50.2
Size of baby		
Small	562	7.9
Average	4992	70.6
Large	1522	21.5
Child had diarrhoea in the last 2 weeks (*n* = 7308)		
No	6207	84.9
Yes	1101	15.1
Child had fever in last two weeks (*n* = 7303)		
No	5560	76.1
Yes	1743	23.9
*Household level factors*		
Wealth Index		
Poorest	1566	21.4
Poorer	1747	23.9
Middle	1647	22.5
Rich	1369	18.7
Richest	996	13.6
Source of drinking water		
Protected	3088	42.2
Unprotected	4237	57.8
*Community level factors*		
Type of residence		
Urban	1442	19.7
Rural	5883	80.3
Geographic Zones		
Northern	956	13.1
Eastern	849	11.6
Western	1547	21.1
Southern Highlands	1016	13.9
Lake	1487	20.3
Southern	567	7.7
Central	708	9.7
Zanzibar	196	2.7

^a^ BF + supplements included BF + liquids/juice; BF + other milk and BF+ complementary foods

**Fig. 1:**
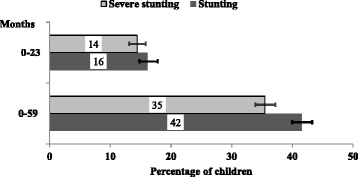
Prevalence of stunting and severe stunting in children aged 0**–**23 and 0**–**59 months

As illustrated in Fig. 1, the prevalence of stunted children aged 0–23 months and 0–59 months was 16 and 42 % respectively. The overall prevalence of severely stunted children aged 0-23 months and 0-59 months was 14 and 35 %, respectively.

### Multivariate analyses

Tables 2 and 3 show the unadjusted and adjusted ORs for the association between stunted and severely stunted children and child-, household- and community-level characteristics of children aged 0-23 and children aged 0-59 months.

**Table 2:** Factors associated with stunting in children aged 0-23 months and 0-59 months

Characteristic	Stunted children 0–23 Months				Stunted children 0–59 Months			
	Unadjusted OR [95 % CI]	*P*	Adjusted OR [95 % CI]	*P*	Unadjusted OR [95 % CI]	*p*	Adjusted OR [95 % CI]	*p*
*Parental factor*								
Maternal working status								
Non-working	1.00				1.00			
Working (past 12 months)	1.57 [1.19–2.07]	0.002			1.23 [1.02–1.49]	0.029		
Maternal education								
Secondary and above	1.00		1.00		1.00		1.00	
Primary	2.08 [1.33–3.26]	0.001	1.82 [1.15–2.86]	0.011	2.26 [1.61–3.18]	<0.0001	1.53 [1.07–2..19]	0.019
No education	2.51 [1.59–3.96]	<0.001	2.26 [1.41–3.60]	0.001	2.54 [1.77–3.64]	<0.0001	1.61 [1.08–2.40]	0.019
Partner's occupation								
Not working	1.00		1.00		1.00			
Agriculture	1.57 [1.06–2.30]	0.023	1.62 [1.05–2.49]	0.027	1.42 [1.21–1.67]	<0.001		
Non agriculture	1.07 [0.72–1.59]	0.712	1.30 [0.80–2.04]	0.233	0.98 [0.72–1.32]	0.889		
Partner's education								
Secondary and above	1.00				1.00			
Primary	2.19 [1.44–3.03]	<0.001			2.08 [1.58–2.14]	<0.001		
No education	1.74 [1.12–2.72]	0.014			2.02 [1.51–2.71]	<0.001		
Mother's age (years)								
15–24	1.00				1.00			
25–34	0..86 [0.70–1.05]	0.145			0.92 [0.80–1.06]	0.246		
35–49	1.20 [0.93–1.55]	0.168			1.10 [0.93–1.31]	0.277		
Mother's age at child’s birth (years)								
20–29	1.00		1.00		1.00			
30–39	1.21 [0.95–1.53]	0.111	1.18 [0.93–1.52]	0.175	1.15 [0.99–1.33]	0.060		
≥ 40	1.47 [0.92–2.35]	0.106	1.66 [1.02–2.70]	0.040	1.13 [0.83–1.54]	0.259		
< 20	1.53 [1.17–2.01]	0.002	1.77 [1.27- 2.46]	0.001	1.28 [1.07–1.53]	0.006		
Marital status								
Currently married	1.00				1.00			
Formerly married^+^	1.27 [0.87–1.84]	0.211			1.18 [0.94–1.48]	0.149		
Never married	0 .78 [0.53–1.15]	0.209			0.80 [0.57–1.11]	0.184		
Birth order								
First-born	1.00				1.00			
2nd -4th	0.96 [0.74–1.24]	0.736			1.04 [0.89–1.22]	0.602		
5+	1.03 [0.77–1.36]	0.854			1.10 [0.92–1.31]	0.302		
Preceding birth interval								
No previous birth	1.00				1.00			
< 24 months	1.09 [0.75–1.59]	0.644			1.25 [0.99–1.58]	0.066		
> 24 months	0.97 [0.75–1.24]	0.792			1.04 [0.89–1.20]	0.651		
Place of delivery								
Home	1.00				1.00			
Health facility	0.81 [0.65–1.00]	0.053			0.76 [0.65–0.88]	<.0001		
Type of delivery assistance								
Health professional	1.00				1.00			
Traditional birth attendant	1.44 [ 1.09–1.91]	0.010			1.55 [1.28–1.88]	<0.001		
Relatives or other	1.28 [0.99–1.64]	0.052			1.33 [1.13–1.57]	0.001		
No one	0.61 [0.32–1.16]	0.130			0.96 [0.68–1.37]	0.831		
Mode of delivery								
Non-caesarean	1.00				1.00			
Caesarean	0.75 [0 .48–1.15]	0.188			0.70 [0.51–0.98]	0.035		
Timing of postnatal check-up								
No check-ups^&^	1.00				1.00			
0-2 days	0.95 [0.73–1.25]	0.729			0.85 [0.70–1.03]	0.101		
3-6 days	1.04 [0.67–1.59]	0.874			0.99 [0.72–1.35]	0.927		
7 + days	0.71 [0.51–0.98]	0.040			0.73 [0.57–0.92]	0.011		
Antenatal clinic visits								
None	1.00				1.00		1.00	
1-3.	0.62 [0.39–0.98]	0.043			0.75 [0.65–0.87]	<0.001	0.78 [0.65–0.95]	0.017
4+	0.59 [0.38–0.92]	0.020			0.71 [0.61–0.84]	<0.001	0.82 [0.65–1.02]	0.080
Maternal BMI (kgm^−2^)								
> 18.5	1.00				1.00		1.00	
< 18.5	1.54 [1.17–2.03]	0.002			1.46 [1.21–1.77]	<0.001	1.38 [1.12–1.69]	0.002
Child BF status								
Exclusive BF	1.00				1.00		1.00	
BF + water	0.94 [0.59–1.70]	0.836			1.04 [0.68–1.59]	0.869	1.09 [0.71–1.67]	0.668
BF + supplements	2.11 [1.51, 2.94]	<0.001			1.20 [0.98–1.46]	0.076	1.26 [1.03–1.53]	0.022
No BF	5.07 [3.40–7.56]	<0.001			1.69 [1.38–2.06]	<0.001	2.02 [1.65–2.46	<0.001
Mother is literate								
No	1.00		1.00		1.00			
Yes	0.93 [0.77–1.12]	0.450	1.36 [1.03–1.82]	0.032	0.82 [0.72–0.93]	0.003		
Mother read newspaper								
No	1.00				1.00			
Yes	0.93 [0.70–1.24]	0.625			0.87 [0.71–1.07]	0.183		
Mother listened to the radio								
No	1.00				1.00			
Yes	1.06 [0.87–1.29]	0.537			0 .92 [0.81–1.04]	0.190		
Mother watched television								
No	1.00				1.00			
Yes	0.79 [0.57–1.08]	0.141			0.63 [0.50–0.81]	<0.001		
*Child level factors*								
Child’s age	1.11 [1.09–1.13]	<0.001	1.11[1.10–1.13]		1.01 [1.01–1.02]	<0.001		
Sex of baby								
Female	1.00		1.00		1.00		1.00	
Male	1.42 [1.17–1.73]	<0.001	1.66 [1.34–2.06]	<0.001	1.36 [1.21–1.52]	<0.001	1.39 [1.23–1.58]	<0.001
Size of baby								
Large	1.00		1.00		1.00		1.00	
Average	1.30 [1.03–1.64]	0.028	1.39 [1.09–1.77]	0.007	1.23 [1.07–1.40]	0.002	1.18 [1.03–1.34]	0.015
Very small	1.93 [1.33–2.81]	<0.001	2.23 [1.47–3.38]	<0.001	1.82 [1.45–2.29]	<0.001	1.92 [1.52–2.43]	<0.001
Child had diarrhoea (past 2 weeks)								
No	1.00				1.00			
Yes	1.11 [0..87–1.41]	0.407			1.03 [0.87–1.22]	0.715		
Child had fever in (past two weeks)								
No	1.00				1.00			
Yes	1.19 [0.96–1.46]	0.115			1.02 [0.88–1.18]	0.754		
*Household level factors*								
Household wealth index								
Richest	1.00				1.00		1.00	
Richer	1.35 [0.89–2.04]	0.155			1.68 [1.25–2.25]	0.001	1.40 [1.03–1.89]	0.030
Middle	1.49 [0.99–2.26]	0.057			2.06 [1.55–2.74]	<0.001	1.67 [1.23–2.28]	0.001
Poorer	1.49 [0.99–2.25]	0.057			2.12 [1.58–2.83]	<0.001	1.81 [1.34–2.45]	<0.001
Poorest	1.85 [1.25–2.74]	0.002			2.48 [1.87–3.29]	<0.001	1.95 [1.43–2.65]	<0.001
Source of drinking water								
Protected	1.00		1.00		1.00		1.00	
Unprotected	1.48 [1.19–1.84]	<0.001	1.33 [1.04–1.70]	0.020	1.42 [1.23–1.63]	<0.001	1.26 [1.08–1.46]	0.002
*Community level factors*								
Type of residence								
Urban	1.00				1.00			
Rural	1.40 [1.06–1.85]	0.019			1.76 [1.43–2.17]	<0.001		
Geographic Zones								
Northern	1.00				1.00			
Eastern	0.84 [0.51–1.37]	0.486			0.62 [0.44–0.86]	0.005		
Western	1.35 [0.96–1.90]	0.085			0.95 [0.75–1.19]	0.640		
Southern Highlands	1.59 [1.05–2.39]	0.026			1.39 [1.01–1.90]	0.043		
Lake	0.93 [0.67–1.28]	0.654			0.83 [0.65–1.05]	0.124		
Southern	1.15 [0.78–1.71]	0.482			1.08 [0.85–1.38]	0.527		
Central	1.55 [1.09–2.19]	0.014			1.30 [0.99–1.70]	0.063		
Zanzibar	0.81 [0.58–2.18]	0.222			0.55 [0.43–0.71]	<0.001		

^&^(including missing)

^+^ (divorced/separated /widowed)

**Table 3:** Factors associated with severe stunting in children aged 0–23 months and 0–59 months

Characteristic	Severely stunted children 0–23 Months				Severely stunted children 0–59 Months			
	Unadjusted OR [95 % CI]	*P*	Adjusted OR [95 % CI]	*p*	Unadjusted OR [95 % CI]	*p*	Adjusted OR [95 % CI]	*p*
*Parental factor*								
Maternal working status								
Non-working	1.00				1.00			
Working (past 12 months)	1.15 [0.77–1.70]	0.497			1.05 [0.78–1.40]	0.761		
Maternal education								
Secondary and above	1.00		1.00		1.00		1.00	
Primary	3.44 [1.68–7.02]	0.001	3.63 [1.58–8.28]	0.002	3.28 [2.04–5.27]	<0.001	1.95 [1.12–3.41]	0.017
No education	4.41 [2.22–8.76]	<0.001	4.86 [2.08–11.35]	<0.001	4.50 [2.74–7.39]	<0.001	2.57 [1.46–4.50]	0.001
Partner's occupation								
Non agriculture	1.00				1.00			
Agriculture	1.43 [1.01–2.03]	0.046			1.50 [1.22–1.84]	<0.001		
Not working	0.73 [0.38–1.41]	0.352			1.08 [0.72–1.61]	0.721		
Partner's education								
Secondary and above	1.00				1.00		1.00	
Primary	1.99 [1.16–3.44]	0.013			2.57 [1.80–3.66]	<0.001	1.79 [1.19–2.71]	0.005
No education	1.87 [1.03–3.40]	0.041			2.92 [1.94–4.39]	<0.001	1.71 [1.07–2.72]	0.022
Mother's age								
15–24 years	1.00				1.00			
25–34 years	0.98 [0.73–1.30]	0.873			1.14 [0.92–1.42]	0.212		
35-49 years	1.46 [0.99–2.14]	0.054			1.27 [0.96–1.66]	0.089		
Mother's age at birth								
< 19 years	1.00				1.00			
20–29 years	0.61 [0.43–0.88]	0.009			0.87 [0.67–1.12]	0.293		
30–39 years	0.97 [0.64–1.47]	0.893			1.08 [0.81–1.45]	0.597		
40 and above	0.88 [0.44–1.76]	0.726			0.93 [0.57–1.52]	0.774		
Marital status								
Currently married	1.00				1.00			
Formerly married^+^	1.49 [0.93–2.39]	0.099			1.33 [0.98–1.79]	0.064		
Never married	0.60 [0.33–1.10]	0.101			0.80 [0.52–1.22]	0.301		
Birth order								
First-born	1.00				1.00			
2nd -4th	0.94 [0.65–1.35]	0.734			1.24 [0.98–1.56]	0.074		
5 or more	1.06 [0.71–1.57]	0.781			1.34 [1.02–1.72]	0.034		
Preceding birth interval								
No previous birth	1.00				1.00			
< 24 months	1.28 [0.80–2.04]	0.296			1.52 [1.15–2.02]	0.003		
> 24 months	0.94 [0.65–1.34]	0.721			1.23 [0.97–1.54]	0.081		
Place of delivery								
Home	1.00				1.00			
Health facility	0.68 [0.52–0.91]	0.010			0.68 [0.57–0.83]	<0.001		
Mode of delivery								
Non-caesarean	1.00				1.00			
Caesarean	0.77 [0.34–1.72]	0.520			0.82 [0.49–1.35]	0.435		
Type of delivery assistance								
Health professional	1.00				1.00		1.00	
Traditional birth attendant	1.70 [1.19–2.42]	0.003			1.88 [1.46–2.43]	<0.001	1.51 [1.15–1.99]	0.003
Relatives and other	1.57 [1.14–2.19]	0.006			1.57 [1.27–1.94]	<0.001	1.34 [1.06–1.70]	0.014
No one	0.89[0.34–2.34]	0.816			0.79 [0.47–1.33]	0.385	0.78 [0.44–1.39]	0.409
Antenatal clinic visits								
4+	1.00		1.00		1.00			
1-3.	1.22 [0.90–1.66]	0.179	1.23[0.89–1.67]	0.199	1.25 [1.01–1.54]	0.043		
None	2.62 [1.57–4.38]	<0.001	2.01[1.17–3.46]	0.012	1.38 [1.11–1.72]	0.004		
Timing of postnatal check-up								
No check-ups^&^	1.00				1.00			
0-2 days	0.94 [0.66–1.34]	0.726			0.82 [0.64–1.06]	0.124		
3-6 days	1.11 [0.64–1.95]	0.703			1.11 [0.70–1.72]	0.684		
7 + days	0.75 [0.48–1.17]	0.207			0.84 [0.63–1.12]	0.239		
Maternal BMI								
> 18.5 (kg/m^2^)	1.00		1.00		1.00		1.00	
<= 18.5 (kg/m^2^)	1.77 [1.20–2.62]	0.004	1.59 [1.05–2.41]	0.028	1.67 [1.28–2.19]	<0.001	1.50 [1.11–2.02]	0.008
Child BF status								
Exclusive BF	1.00				1.00			
BF + water	0.84 [0.29–2.45]	0.756			0.71 [0.34–1.36]	0.303		
BF + supplements	1.74 [1.04–2.92]	0.035			0.96 [0.76–1.27]	0.772		
No BF	3.07 [1.80–5.23]	<0.001			1.16 [0.89–1.51]	0.270		
Mother was literate								
No	1.00				1.00			
Yes	0.85 [0.65–1.09]	0.209			0.71 [0.60– 0.85]	<0.001		
Listening to radio								
No	1.00				1.00			
Yes	0.89 [0.67–1.18]	0.445			0.80 [0.67–0.94]	0.009		
Mother read newspaper/magazine								
No	1.00				1.00			
Yes	0.81 [0.55–1.19]	0.292			0.82 [0.64–1.04]	0.114		
Mother watched TV								
No	1.00				1.00			
Yes	0.79 [0.51–1.21]	0.278			0.67 [0.50–0.90]	0.007		
*Child level factors*								
*Child’s age*	1.06 [1.09–1.11]	<0.001	1.09 [1.07–1.12]	<0.001	1.00 [1.00–1.01]	0.002		
Sex of baby								
Female	1.00		1.00		1.00		1.00	
Male	1.46 [1.13–1.89]	0.003	1.63 [1.22–2.16]	0.001	1.36 [1.17–1.58]	<0.001	1.45 [1.23–1.72]	<0.001
Size of baby								
Large	1.00		1.00		1.00		1.00	
Average	1.60 [1.15–2.24]	0.005	1.65 [1.18–2.31]	0.004	1.55 [1.25–1.90]	<0.001	1.48 [1.19–1.84]	<0.001
Very small	3.06 [1.77–5.29]	<0.001	3.26 [1.80–5.90]	<0.001	2.54 [1.88–3.43]	<0.001	2.64 [1.92–3.63]	<0.001
Child had diarrhoea in the last 2 weeks								
No	1.00				1.00			
Yes	1.06 [0.75–1.51]	0.738			1.15 [0.92–1.42]	0.212		
Child had fever in last 2 weeks								
No	1.00				1.00			
Yes	1.19 [0.87–1.62]	0.272			0.99 [0.78–1.25]	0.941		
*Household level factors*								
Wealth Index								
Poorest	1.00				1.00			
Poorer	1.01 [0.56–1.82]	0.979			1.50 [0.96–2.35]	0.074		
Middle	1.18 [0.64–2.18]	0.585			1.77 [1.19–2.63]	0.005		
Rich	1.51 [0.86–2.65]	0.147			2.29 [1.54–3.41]	<0.001		
Richest	1.52 [0.85–2.75]	0.159			2.39 [1.59–3.58]	<0.001		
Source of drinking water								
Protected	1.00		1.00		1.00		1.00	
Unprotected	1.58 [1.15–2.18]	0.005	1.50 [1.05–2.14]	0.025	1.58 [1.29–1.94]	<0.001	1.22 [1.13–1.79]	0.003
*Community level factors*								
Type of residence								
Rural	1.00		1.00		1.00			
Urban	1.06 [0.74–1.53]	0.734	1.52 [1.02–2.27]	0.040	1.54 [1.16–2.05]	0.003		
Geographic Zones								
Northern	1.00				1.00		1.00	
Eastern	0.81 [0.40–1.60]	0.530			0.65 [0.35–1.03]	0.065	0.74 [0.43–1.27]	0.281
Western	1.09 [0.69–1.73]	0.692			0.70 [0.48–1.02]	0.065	0.64 [0.42–0.95]	0.028
Southern Highlands	1.28 [0.76–2.15]	0.351			1.15 [0.77–1.71]	0.473	1.13 [0.75–1.71]	0.546
Lake	0.78 [0.45–1.37]	0.389			0.74 [0.48–1.16]	0.199	0.73 [0.46–1.13]	0.164
Southern	0.98 [0.57–1.70]	0.961			0.97 [0.66–1.43]	0.888	0.96 [0.65–1.43]	0.876
Central	1.53 [0.91–2.56]	0.105			1.31 [0.89–1.92]	0.165	1.15 [0.77–1.70]	0.484
Zanzibar	0.93 [0.57–1.50]	0.765			0.59 [0.40–0.86]	0.007	0.80 [0.52–1.24]	0.327

^&^(including missing)

^+^ (divorced/separated /widowed)

### Risk factors for stunting

Table 2 shows factors that posed risk to stunting among children aged 0-23 months and those aged 0-59 months. Increased child age was found to be statistically associated with stunted children aged 0-23 months. The risk of stunting was significantly higher among male children compared to females for both age brackets. Children who were perceived by their mothers to be very small or small at birth were significantly more likely to be stunted than those who were perceived to be large. Babies delivered by younger mothers (aged less than 20 years) were significantly more likely to be stunted compared to those delivered by mothers aged 20–29 years. The odds for stunting among children of both age brackets increased significantly among those who lived in households with no access to potable water and for those whose fathers had limited or no schooling and worked in an agricultural industry. Children who were delivered at home, who were delivered by traditional birth attendants (TBAs), whose mothers did not have any antenatal clinic visits and those whose mothers had a Body Mass index (BMI) of less than 18.5kgm^−2^ were significantly more likely to be stunted. The risk of stunting was also found to be significantly high among children who were given supplements in addition to breast milk and as well as those who were non-breastfed. Other risk factors associated with stunting were rural children, children from the poorest households, children whose mothers were illiterate, in paid employment and resided in the Southern Highlands zone of Tanzania.

### Risk factors for severe stunting

Table 3 shows the risk factors associated with severe stunting among children aged 0-59 months. Male children and babies perceived by their mothers to be small at birth were significantly more likely to be severely stunted compared to females and babies perceived to be of medium or large size at birth. The risk of severe stunting was significantly higher among children whose parents had no schooling and were illiterate. Children from poorest households, those who resided in urban areas and in the Northern zone of Tanzania were significantly more likely to become severely stunted. The risk of severe stunting was significantly higher among children who were delivered at home by Traditional Birth Attendants (TBAs) and whose mothers did not attend any antenatal clinics. Children who were 5^th^-born or higher, children who were perceived by their mothers to be small at birth and those from poorest households with no potable drinking water were significantly associated with severe stunting (Table 3).

## Discussion

The present paper was designed to determine factors associated with stunting and severe stunting among Tanzania children aged 0-59 months. The main risk factors for stunting in the study were: age of the child, child’s sex, maternal level of educational, perceived size of the child at birth, mother’s age at child’s birth, place of delivery, type of birth delivery assistance, maternal BMI and breastfeeding status of a child. Factors associated with severe stunting included: sex of the child, parent’s level of education and literacy, household wealth index, place of delivery and type of delivery assistance. Birth order of the child, perceived size of the baby at birth, source of drinking water and geographical region were also factors significantly associated with severe stunting.

The main strengths of our study were that it used a nationally-representative survey data and applied appropriate statistical adjustments for the cluster sampling design in the analysis. Our analysis was able to determine the most vulnerable age group and the modifiable characteristics that affected stunting in a large sample size. One key limitation, however, was that we could not establish the cause and effect relationships; because of the cross-sectional nature of the study design. In addition, although a comprehensive set of variables were used in our analysis, residual confounding from unmeasured covariates could not be ruled out.

Our study found that children in the 0-23 month age bracket had a significantly lower risk of being stunted compared to those in the older age bracket (0-59 months). Similar findings were reported by a recent study [18]. This finding may be due to the protective effect of breastfeeding, since almost all children in Tanzania are breastfed and most of them continue to be breastfed throughout the first year of their life [19]. The high risk of stunting observed beyond the 0-23 months-period may be linked to inappropriate food supplementation during the weaning period [20].

Children whose parents had no schooling were found to have a relatively higher risk of being stunted or severely stunted. This finding is consistent with those found in previous studies [20–23] in which stunting and severe stunting were positively associated with lower levels of parental education, which may be explained by the resulting limited family income and the consequent inadequate individual care and attention given to the child. Educated mothers would be more conscious about their children’s health. Children whose mothers perceived them to be small or very small at birth were found to be at a relatively higher risk of being stunted compared to other children in this age group. A similar association between birth weight, which has been found to be a measure of perceived size of the baby [24] and later risk for stunting has previously been documented in other low-income countries [15, 25]. Such children were found to be associated with severe stunting, consistent with previous other studies [25–27]. Small newborns from less affluent areas thus do not seem to demonstrate marked catch-up growth during infancy. As the prevalence of low birth weight (<2500 g) is as high as 16 % in Tanzania [19], prevention of intrauterine growth retardation and preterm births must form one cornerstone in the population level management strategy for infant stunting. Assessment of the size of the baby at birth may be important for health care providers since this can be used to identify the risk of stunting among children in order to take necessary measures.

In this current study, male children aged 0-59 months were found to have a higher risk of being stunted or severely stunted, compared to females. This finding is consistent with a finding reported from a meta-analysis of sixteen demographic and health surveys of ten countries in sub-Saharan Africa, in which male children were found to be consistently more likely to become stunted compared to their female counterparts [28]. A recent study also associated male children with severe stunting [26]. These sex differences in stunting and severe stunting could be explained by behavioural patterns employed by communities such as favouritism which may involve dietary intakes towards daughters. In a previous study, it was reported that males were given supplemental foods earlier, were fed larger quantities of supplemental foods and had higher rates of diarrhoea compared to females [29].

Our study found that children whose mothers had no schooling were more likely to become stunted compared to those whose mothers had secondary education or higher. This reflects the importance of education for mothers in regard to the development of healthy children, as reported by previous studies [10].

In the present study, children from poorest households were found to have a significantly higher risk of being stunted compared to those from the middle-income, rich and richest households. The effect of wealth on stunting can be explained by its importance in the purchase of food and consumer goods that promote and protect the health of children. Various studies have observed a positive association between low income and malnutrition [10, 30, 31], which often leads to stunting.

In our analyses, children born to mothers of low BMI were more likely to be severely stunted compared to those born to mothers with higher BMI. Previous studies have associated stunting with maternal factors and in particular the mother's poor nutritional status before conception and poor nutrition during pregnancy [15, 32, 33]. Sufficient weight gain during pregnancy is particularly important since it accounts for a large proportion of foetal growth retardation.

The risk of stunting was found to be significantly higher among children who were no longer breastfeeding and those who were breastfed longer than 12 months. A recent study in Ecuador [34] revealed that children who were stunted made up 30 % of those exclusively breastfed for less than or equal to 6 months, 23.3 % of the children exclusively breastfed for between 6-12 months and 27.7 % of those exclusively breastfed for 12 months or longer. Although the WHO recommends that women exclusively breastfeed their children for 6 months, there has been evidence that breastfeeding alone may not adequately meet the nutritional requirements of a 6 month-old baby [35]. If this is indeed the case, it may be likely that exclusively breastfeeding for 12 months or longer does not provide enough energy for growing babies in Tanzania. Our study also found that infants who were not breastfed were significantly associated with stunting and severe stunting among children from both age groups. This finding is consistent with previous studies [36–38].

In our study, we found that children born to relatively younger mothers (<20 years) had a significantly higher risk of being stunted. This may be attributed to the fact that such mothers would not have the requisite experience or knowledge to provide the child with the proper care. However, a previous study reported that mother’s age at pregnancy is not a predictor of stunting [39].

Being born at home and delivered by TBAs were found to be significant risk factors to severe stunting for children aged 0-59 months. This finding is consistent with a previous study in rural Malawi [26]. Mothers of such children may not have had any contacts with trained medical professionals to receive proper advice on appropriate child feeding practices.

## Conclusions

This current study has highlighted the individual-, household- and community-level factors associated with stunting and severe stunting among Tanzanian children. The main risk factors included male children, children perceived to be small at birth, children from poorest households with no potable water and those who were born at home with assistance from traditional birth attendants. Our findings indicate the need for interventions at both the individual and community levels. Peer-based community interventions including peer-education, where older and more experienced women could educate these young mothers about appropriate child feeding practices aimed at long-term prevention of stunting and severe stunting in Tanzania are required to improve child health. At the individual level, emphasis should be placed on educating mothers and particularly young mothers regarding health and child feeding practices including safe sources of drinking water for their children.

## Abbreviations

WHO: World Health Organization
TDHS: Tanzania Demographic and Health Survey
SD: standard deviation
TBAs: Traditional Birth Attendants
BMI: Body Mass index
OR: Odds ratio
CI: Confidence interval
